# Spatiotemporal analysis and socioeconomic determinants of pediatric lymphoid leukemias mortality in Brazil from 2000 to 2021: an ecological study

**DOI:** 10.1016/j.lana.2026.101557

**Published:** 2026-07-09

**Authors:** Bárbara Sarni Sanches, Isadora Lima Oliveira, Ciana Duque Estrada Botelho, Nathalia Lopez Duarte, Ursula Berger, Marcelo Gerardin Poirot Land

**Affiliations:** a Faculty of Medicine, Federal University of Rio de Janeiro (UFRJ), Rio de Janeiro, RJ, Brazil; b National Institute of Science and Technology in Childhood Cancer Biology and Pediatric Oncology (INCT BioOncoPed), Porto Alegre, RS, Brazil; c Institute for Medical Information Processing, Biometry and Epidemiology, Ludwig-Maximilian University of Munich, Munich, BY, Germany; d Department of Pediatrics, Martagão Gesteira Institute for Childcare and Pediatrics, Rio de Janeiro, RJ, Brazil

**Keywords:** Pediatric cancer, Lymphoid leukemias, Socioeconomic disparities, Socioeconomic determinants

## Abstract

**Background:**

Leukemias are the most frequent pediatric cancers, and socioeconomic factors influence their outcomes. In Brazil, regional disparities in pediatric lymphoid leukemias (LL) mortality have been reported. Their determinants require further investigation. We aimed to describe pediatric LL mortality and its association with socioeconomic, demographic, and health system characteristics in Brazil.

**Methods:**

This ecological study included the Brazilian state population aged 0–19 years from 2000 to 2021. Crude and age-specific mortality rates were the primary outcome. Annual percentage change estimated mortality trends. Eleven socioeconomic characteristics were grouped in two components using principal component analysis (PCA): “development index” and “Primary Health Care (PHC) index”. Generalized additive mixed models explored their associations with mortality.

**Findings:**

Pediatric LL mortality increased in Brazil, mainly in North and Northeast (NE). South and Southeast showed stable or declining rates. The development index association with age-specific mortality rates (ASMR) increase was crescent in worse socioeconomic contexts and decrescent in better ones. The PHC index showed a non-significant association with ASMR decreases in NE states.

**Interpretation:**

Regional disparities in pediatric LL mortality were associated with socioeconomic inequalities. More vulnerable states experienced mortality increases and delayed benefits of economic and health system development. PHC expansion alone was insufficient to overcome disparities. Strengthening health workers training and treatment capacity is essential.

**Funding:**

Coordenação de Aperfeiçoamento de Pessoal de Nível Superior, Conselho Nacional de Desenvolvimento Científico e Tecnológico, Fundação de Amparo à Pesquisa do Estado do Rio de Janeiro, INCT BioOncoPed, and the International Society of Paediatric Oncology.

## Resumo

**Introdução:**

As leucemias são os cânceres pediátricos mais frequentes, e fatores socioeconômicos influenciam seus desfechos. No Brasil, foram descritas disparidades regionais na mortalidade pediátrica por leucemias linfoides (LL). Seus determinantes necessitam melhor elucidação. O objetivo deste estudo foi descrever a mortalidade por LL pediátrica e sua associação com características socioeconômicas, demográficas e do sistema de saúde no Brasil.

**Métodos:**

Este estudo ecológico incluiu a população dos estados brasileiros de 0–19 anos entre 2000–2021. As taxas de mortalidade brutas e específicas por idade foram o desfecho primário. A variação percentual anual estimou as tendências de mortalidade. Onze características socioeconômicas foram agrupadas em dois componentes por análise de componentes principais (ACP): “índice de desenvolvimento” e “índice de Atenção Primária à Saúde (APS)”. Modelos mistos aditivos generalizados exploraram suas associações com a mortalidade.

**Resultados:**

A mortalidade pediátrica por LL aumentou no Brasil, principalmente no Norte e Nordeste (NE). Sul e Sudeste apresentaram taxas estáveis ou em declínio. A associação do índice de desenvolvimento com o aumento das taxas de mortalidade específicas por idade (TMEI) foi crescente em contextos socioeconômicos menos favorecidos e decrescente nos mais favorecidos. O índice de APS apresentou uma associação não significativa com a redução das TMEI nos estados do NE.

**Interpretação:**

As disparidades regionais na mortalidade pediátrica por LL estiveram associadas a desigualdades socioeconômicas. Estados mais vulneráveis sofreram maior aumento de mortalidade e benefícios tardios do desenvolvimento econômico e do sistema de saúde. A expansão da APS isoladamente foi insuficiente para superar as disparidades. Fortalecer a capacitação dos profissionais de saúde e a capacidade de tratamento é essencial.

**Financiamento:**

Coordenação de Aperfeiçoamento de Pessoal de Nível Superior, Conselho Nacional de Desenvolvimento Científico e Tecnológico, Fundação de Amparo à Pesquisa do Estado do Rio de Janeiro, INCT BioOncoPed e International Society of Paediatric Oncology.

**Research in context**

**Evidence before this study**

We searched PubMed, Scopus, Web of Science, SciELO, and Google Scholar for studies published from Jan 1, 2000, to December 22, 2025, without language restrictions, using combinations of the terms “childhood leukemia”, “pediatric leukemia”, “lymphoid leukemia”, “acute lymphoblastic leukemia”, “mortality”, “survival”, “Brazil”, “Latin America”, “socioeconomic determinants”, “social inequalities”, “regional disparities”, “health system access”, and “primary health care”. We also reviewed reports from the World Health Organization (WHO), the Pan-American Health Organization (PAHO), the Brazilian National Institute for Cancer (INCA), the Brazilian Institute of Geography and Statistics (IBGE), and the Brazilian Ministry of Health. Previous studies consistently reported large survival gaps between high-income and low- and middle-income countries, as well as regional inequalities in childhood leukemia outcomes in Brazil. Earlier Brazilian analyses showed heterogeneous mortality trends across regions and inverse correlations between socioeconomic indicators and leukemia mortality. However, most studies relied on single indicators, limited time periods, or descriptive approaches, and did not examine how demographic, socioeconomic, health system, and data-quality dimensions dynamically shape age-specific lymphoid leukemia mortality patterns over space and time in Brazil.

**Added value of this study**

This study extends previous evidence by showing that regional disparities in pediatric lymphoid leukemia mortality in Brazil are not fully captured by isolated socioeconomic indicators. By integrating socioeconomic, demographic, health-system, and data-quality characteristics into composite indices, the study identifies a context-dependent pattern in which these aspects may coincide with increasing mortality in less advantaged settings, while becoming associated with mortality reductions after higher structural capacity is reached. These findings help explain why improvements in economic indicators or health access alone may not translate into better pediatric leukemia outcomes across more vulnerable Brazilian regions. The “development index” adds a different dimension to the Human Development Index (HDI), and may be further used as a health adjusted development index for monitoring purposes within the Brazilian context.

**Implications of all the available evidence**

Persistent regional disparities in pediatric lymphoid leukemia mortality in Brazil are linked to structural socioeconomic and health-system inequalities rather than health care access alone. Policies focused exclusively on expanding primary health care are unlikely to reduce mortality without parallel investments in diagnostic capacity, referral pathways, specialized treatment infrastructure, physician workforce, data quality, and socioeconomic development. Targeted, region-specific strategies are needed to address subnational inequities in childhood cancer outcomes. Future studies integrating survival, treatment quality, diagnostic timeliness, treatment abandonment, and genomic data may clarify individual mechanisms underlying these disparities and inform more effective national and subnational policies.

## Introduction

Lymphoid leukemias (LL) are the most frequent pediatric cancers, accounting for one-third of all diagnosed cancer cases in children and adolescents worldwide.^1^, ^2^, ^3^, ^4^ Since 1975, the incidence of pediatric cancer has increased in high-income countries (HIC), while mortality has steadily declined, largely due to advances in leukemias treatment. In contrast, low- and middle-income countries (LMIC) have not experienced comparable reductions in mortality rates.^4^^,^^5^ The epidemiologic transition model has been used to explain such shifts in mortality patterns over time, describing the change of predominance from infectious to chronic diseases, driven by demographic and socioeconomic changes. However, this model was originally developed based on European populations and may not fully account for the complex interplay of factors that shape disease outcomes in LMICs.^6^^,^^7^

While remission rates for LL exceed 90% in HICs, they remain below 30% in LMICs, resulting in significantly worse survival outcomes in these settings.^4^^,^^8^ It is expected that delays in diagnosis and treatment and lack of early detection programs directly contribute to these observed disparities.^9^, ^10^, ^11^, ^12^ Evidence from HICs shows that disparities in pediatric LL outcomes persist despite universal healthcare systems, highlighting the role of factors beyond healthcare availability, including immigration status, health behaviors, and individual or regional socioeconomic conditions.^8^^,^^13^^,^^14^

In contexts of limited or unevenly distributed resources, identifying and understanding both socioeconomic barriers and healthcare system’s weaknesses are essential for addressing inequalities in childhood LL outcomes.^15^^,^^16^ Brazil is a continent-sized country marked by high levels of inequality across its 27 states (or federative units). States in the South and Southeast consistently exhibit the highest human development index (HDI), literacy rates, and gross domestic product (GDP) per capita of the country, while states in the North and Northeast have the lowest indicators.^17^ In Brazil, 7560 new cancer cases are estimated for children and adolescents in 2026, with higher incidence rates in the South and Southeast regions. In 2023, 2326 pediatric cancer deaths were reported, with different regional disparities also described, and higher rates in the North and Northeastern regions, while the lowest rate was observed in Distrito Federal.^18^, ^19^, ^20^, ^21^ The underlying factors driving these disparities remain insufficiently understood. This study aimed to describe spatiotemporal patterns of pediatric LL mortality in Brazil and examine their association with socioeconomic, demographic, and health-system characteristics to identify factors that may contribute to regional disparities in childhood cancer outcomes within the Brazilian context.

## Methods

### Study design and data sources

This is an ecological study which included the pediatric population (aged zero to 19 years) from each Brazilian state (27 federative units) for the period of 2000–2021. This population was divided into four age groups: zero to four, five to nine, 10 to 14, and 15 to 19 years old. The number of deaths by specific cause according to ICD-10 was obtained from the Brazilian Ministry of Health’s open-access database for different states and age groups considering the ICD-10 code C91 (lymphoid leukemias).^22^^,^^23^ This information is reported by the Brazilian Mortality Information System (SIM) and is based on the death certificates issued in each state for each age group during the study period, with LL recorded as the cause of death. The population size was sourced from the Brazilian Institute of Geography and Statistics (IBGE).^24^ This study is reported in accordance with the Strengthening the Reporting of Observational Studies in Epidemiology (STROBE) guidelines.^25^

Incidence and survival rates were not assessed in this study due to the limitations of cancer incidence data in Brazil. Population-Based Cancer Registries, which form the basis of incidence data, are unevenly distributed across the country, particularly in the North and Northeastern regions, where data are sparse or absent. However, SIM is a nationwide system in which deaths from all causes, including cancer, must be officially registered, both for the private and public health system, even if the patient was never formally registered in an incidence database.^26^ The choice to conduct analysis by states was made due to the rarity of pediatric LL. Conducting the analysis for smaller units (such as municipalities, health regions or neighbourhoods) could provide more precise socioeconomic and health information, but could potentially reduce the power of analysis due to the smaller number of observations. Stratification by sex would have also reduced the number of observations within each subgroup, resulting in unstable estimates. Therefore, data from both sexes were analysed together. As this ecological study relied on aggregated mortality and population data, information on gender, race, ethnicity and individual socioeconomic position was unavailable.

### Socioeconomic determinants

Eleven socioeconomic, demographic and health system regional characteristics were selected based on their possible impact on mortality rates from LL.^9^^,^^27^^,^^28^ While some variables represent characteristics of individuals (e.g., life expectancy), the data are reported in an aggregate form, reflecting the distribution of these traits across the population of each Brazilian state rather than individual-level information. All variables are anonymous and sourced from publicly available databases. Data sources are described in detail in Supplementary Table S4, supplements.

**Fig. 1:**
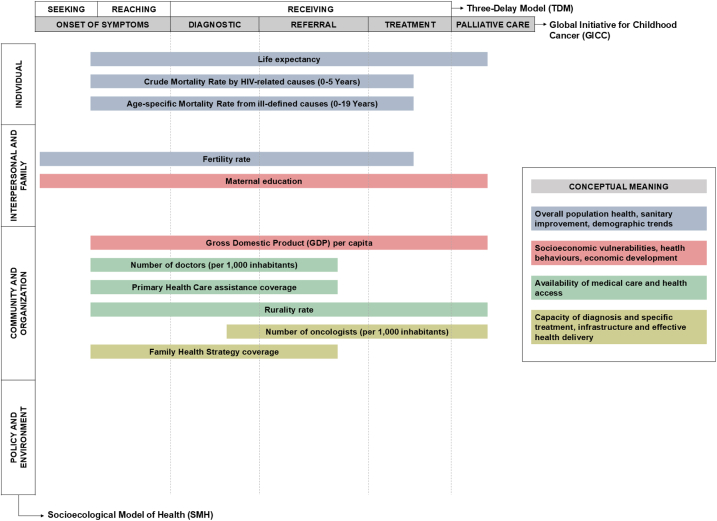
Socioeconomic, demographic and health system-related explanatory variables included in this study, their conceptual meaning and their classification according to the different domains of the Three-Delay Model for Childhood Cancer Care.

The variables were classified using the Three-Delay Model for Childhood Cancer Care proposed by Cotache-Condor et al. (2023), a framework specifically designed for pediatric cancer in LMICs that builds on three well-established models used to understand barriers and determinants in health care.^28^ The Three-Delay Model is used to identify points at which delays occur in seeking, reaching, or receiving care; the WHO’s CureAll framework outlines the critical stages in the management of childhood cancer, from symptom onset to diagnosis, treatment, and palliative care; and the Socioecological Model of Health characterizes the sphere of influence of each determinant, ranging from individual and family to community and policy levels.^4^^,^^29^^,^^30^ By integrating these models, the Cotache-Condor et al. (2023) approach provides a pediatric cancer-specific perspective that allows for the assessment of both the timing and context in which each variable may affect outcomes. Fig. 1 describes the eleven characteristics, their classification and their conceptual meaning.

### Data quality and missing data

We used the proportion of ill-defined deaths in the pediatric population also as a measure of registry quality, representing the percentage of pediatric deaths for which the cause was not specified in sufficient detail to allow classification as a specific disease or condition. A proportion of ill-defined deaths below 6% is considered a low rate and indicates a good data registry quality.^27^

Some variables had missing values due to the absence of records, particularly in the earlier years of the study. The affected variables were rurality rate (50% missing, n = 594), number of doctors (22.7%, n = 594), number of oncologists and hematologists (22.7%, n = 594), GDP per capita (9%, n = 594), Family Health Strategy (FHS) coverage (31.8%, n = 594), and Primary Health Care (PHC) coverage (31.8%, n = 594). To address this, we performed curve estimation for each variable considering a range of regression models (linear, logarithmic, inverse, quadratic, cubic, exponential, and logistic) following the stochastic regression-based imputation model.^31^ For each variable and state, all models were fitted and compared, and the simplest model among those with the highest R^2^ was selected to generate imputed values. This analysis was conducted using IBM SPSS Statistics Software version 20.

### Statistical analysis

The primary outcomes were the crude and the age-specific mortality rates from LL among individuals aged 0–19 years in each Brazilian state from 2000 to 2021. The main analytical hypothesis was that state-level socioeconomic, demographic, health-system, and data-quality characteristics are associated with disparities in pediatric LL mortality. Because this was a nationwide ecological study, no sample-size calculation was performed.

For time trend analysis, we calculated the crude mortality rate (CMR) from LL in the pediatric population (0–19 years) using the observed number of deaths by states and calendar years. We then calculated the annual percentage change (APC) of the CMR in Brazil and its five macroregions (North, Northeast, Southeast, South and Midwest). This analysis was conducted using Joinpoint Regression Program Version 6.0.0.

Since the eleven socioeconomic, demographic and health-system related variables included are strongly correlated (Supplementary Fig. S3, supplements), we used principal component analysis (PCA) to reduce dimensionality and extract orthogonal, uncorrelated components from them. Each component represents a linear combination of the original variables, weighted by their contribution to the component. This strategy has the advantage of allowing the components to be interpreted as integrated constructs, capturing broader underlying dimensions of the socioeconomic, demographic, and health system context of each state in a given year, rather than considering each isolated variable.

In a next step, we used dynamic generalized additive mixed models (GAMM) to model age-specific mortality rates (ASMR) from LL for each age group (0–4 y, 5–9 y, 10–14 y and 15–19 y), and to explore the association of PCA factors on these rates. This modelling approach was selected due to GAMMs’ flexibility in accommodating non-linear relationships between covariates and the outcome, while random effects accounted for the hierarchical and longitudinal data structure, allowing for partial pooling across states and age groups and thereby stabilizing estimates in the presence of sparse data.^32^^,^^33^ This analysis was conducted using the “mgcv” package in RStudio Version 2025.05.0 Build 496.

We used negative binomial models to fit the number of deaths, adjusted by an offset for the population size, allowing the model to interpret the outcome as a mortality rate. To account for intra-state correlation due to the longitudinal nature of the data, we employed mixed-effects models including random effects at the state level. The covariates “time” (in calendar years) and the factors derived from the PCA were included as an additive effect, and age groups were included as categorical variables, with zero to 4 years as the reference group. We compared a set of models of different complexity using Akaike Information Criterion (AIC) and R^2^. The choice of the final model was based on this comparison and also in the interpretability of the model terms. The statistical significance of the smooth terms and the contributions of the covariates in the GAMMs was assessed using the Wald test.

Funding sources involved in this study had no role in study design, data collection, data analysis, interpretation or writing of the report.

### Ethical statement

All data used in this study are publicly available through Brazilian governmental open-access repositories (DATASUS, SIM, CNES, IBGE, and SAPS). No identifiable individual-level data were used; therefore, no individual informed consent was required. The study was reviewed and approved by the Ethics Committee of the Martagão Gesteira Institute of Pediatrics and Childcare (IPPMG/UFRJ) under the Certificate of Presentation of Ethical Appreciation number 73303123.8.0000.5264 (approval date: September 12, 2023). This study was conducted in accordance with the principles of the Declaration of Helsinki.^34^

### Role of the funding source

The funders did not have any role in study design, data collection, data analysis, interpretation or writing of this report. The authors were not paid to write this article by any company or agency. The authors had full access to all the data in the study and accept responsibility to submit the manuscript for publication.

## Results

Concerning the data quality assessment, the proportion of ill-defined deaths was higher in states from the North and Northeast regions. These rates showed a marked decline over time in Brazil, both in central tendency and dispersion, suggesting a consistent improvement in the quality of mortality data over the period. From 2007 onwards, the proportion of ill-defined deaths was below 6% in most Brazilian states (Supplementary Fig. S1).

The PCA resulted in two principal components (Table 1), herein also referred to as factors, which explained 70.4% of the total variance of the dataset. Factor one showed high positive weights for maternal education, number of doctors, number of oncologists, GDP per capita, and life expectancy. Conversely, the proportion of ill-defined deaths, fertility, and rurality presented strong negative weights. This means that higher scores of factor one represent states with higher maternal education, greater physician workforce availability, higher GDP per capita, and improved data quality, alongside lower fertility and rurality. Taken together, these patterns suggest that factor one captures demographic and economic transformations, as well as improvements in data quality and growth of physician workforce. Based on this factor’s composition, we named it “development index”.

**Table 1:** Principal components derived from principal component analysis (PCA) and the corresponding loading (weights) for each variable inside the components.

	Variables’ weights	
	F1 (Development index)	F2 (PHC index)
Maternal education	0.924	
Number of doctors	0.885	
Number of oncologists	0.865	
Gross domestic product per capita	0.860	
Life expectancy	0.776	0.228
Ill-defined deaths proportion	−0.761	
Fertility	−0.761	
Rurality	−0.643	0.480
Family health strategy coverage		0.962
Primary health care coverage		0.953
HIV death rate (<5 years)	−0.258	−0.460

Loadings represent the correlation between the original variables and each component, while squared loadings indicate the relative contribution of each variable to the component structure. Variables are grouped according to their highest loading to facilitate interpretation. For clarity, loadings with absolute values < |0.200| are not displayed.

Factor two, in turn, showed high positive weights for the Family Health Strategy (FHS) coverage and Primary Health Care (PHC) coverage. Based on this factor’s composition, we named it “Primary Health Care index”. Higher values of this index indicate better reach and higher effectiveness of the PHC structure in states.

Absolute values of factor one and two in each state and year can be found in Supplementary Table S5.

From 2000 to 2021, a total of 11,130 children and adolescents died from LL in Brazil. In 2000, the country recorded 488 death cases, with a CMR of 0.70 per 100,000. In 2021, the number of deaths remained stable, accounting for 468 cases, while the pediatric population decreased, resulting in an increase in the CMR (CMR = 0.79 per 100,000; Annual Percentage Change (APC) = 0.61, 95% CI: 0.12–1.11, p = 0.014). This increase was more evident in the North and Northeast regions, while South and Southeast regions showed stable or declining rates (Supplementary Table S1). Supplementary Table S6 shows the total number of reported deaths per state during the entire period.

The selected model used to predict age-specific mortality rates (ASMR) and explore their association with PCA-derived factors is summarized in Table 2 and can be defined as:

$$\log\left(E\left[Y_{i,g,y}\right]\right)=\log\left({pop}_{i,g,y}\right)+\beta_{0}+\sum_{k=1}^{3}\beta_{k}.a_{i,k}+\beta_{4}.y+\beta_{5}.{F2}_{i,g,y}+f_{1}\left({F1}_{i,g,y}\right)\;+\;u_{g}$$

Where log is the link function; $E\left[Y_{i,g,y}\right]$ is the expected number of deaths in the age group i, state g and year y; $\log\left({pop}_{i,g,y}\right)$ is the offset function of the population; $\beta_{0}$ is the intercept; $a_{i,k}$ represents the indicator variables for age groups 5–9 y, 10–14 y and 15–19 y (reference = age group 0–4 y); $\beta_{4}$ is the coefficient of year as a fixed effect; $\beta_{5}$ is the coefficient of factor two; $f_{1}\left(.\right)$ is the smooth function of factor one and $u_{g}$ is the random effect of space (states). Incorporating a non-linear relationship between F1 and the outcome improved model performance, while a similar approach for F2 did not yield any substantial benefit.

**Table 2:** Impact of age, calendar year, the development index (factor 1) and the PHC Index (factor 2) on age-specific mortality rates from lymphoid leukemias in the selected generalized additive mixed model (GAMM).

Variable	β	95%-CI	p-value^a^
Age groups			
0–4 y	–	–	–
5–9 y	0.11	0.04, 0.19	0.002^b^
10–14 y	−0.01	−0.08, 0.06	0.80
15–19 y	−0.06	−0.13, 0.02	0.13
Year	0.02	0.01, 0.03	0.002^b^
Factor two	−0.03	−0.08, 0.03	0.40
Factor one (smooth)			<0.001^b^

β = regression coefficients (when linear); CI = Confidence Interval.

a Wald test.

b p < 0.05.

**Fig. 2:**
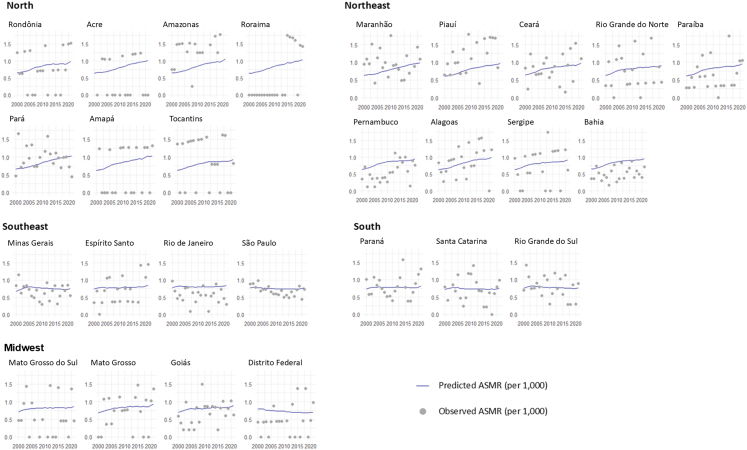
Age-specific mortality rate (ASMR) from lymphoid leukemias in children aged zero to four years in each Brazilian state, grouped in macroregions for representation, from 2000 to 2021. Observed ASMR are represented by grey dots and fitted ASMR are represented by blue lines.

Similar to what was observed in the CMR, nationwide the predicted ASMR from LL increased throughout the study period in all age groups (Supplementary Fig. S2). Increases in mortality were more evident in states from the North and Northeast regions while rates were stable or decreasing in Southern and Southeastern states (Fig. 2). The effect of calendar time (in years) corroborates the observed general increase in mortality (β = 0.02, 95% CI: 0.01–0.03, p = 0.002). Among the age groups, children aged five to nine years experienced a higher mortality compared to the ones aged zero to four years (β = 0.11, 95% CI: 0.04–0.19, p = 0.002).

**Fig. 3:**
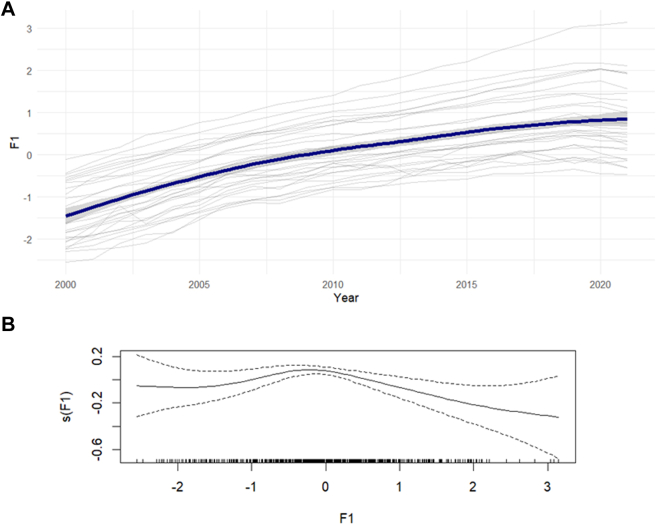
Graphic representations of the development index (factor one, or F1) and its contribution to the predicted age-specific mortality rate (ASMR). **(A)** Observed values of the development index in each state (grey lines) from 2000 to 2021 and the regression line (blue line) representing the general trend of this index over the years. **(B)** Smooth function of the development index (y-axis) for each observed value of this index, which represents its contribution to the ASMR depending on its observed value.

**Fig. 4:**
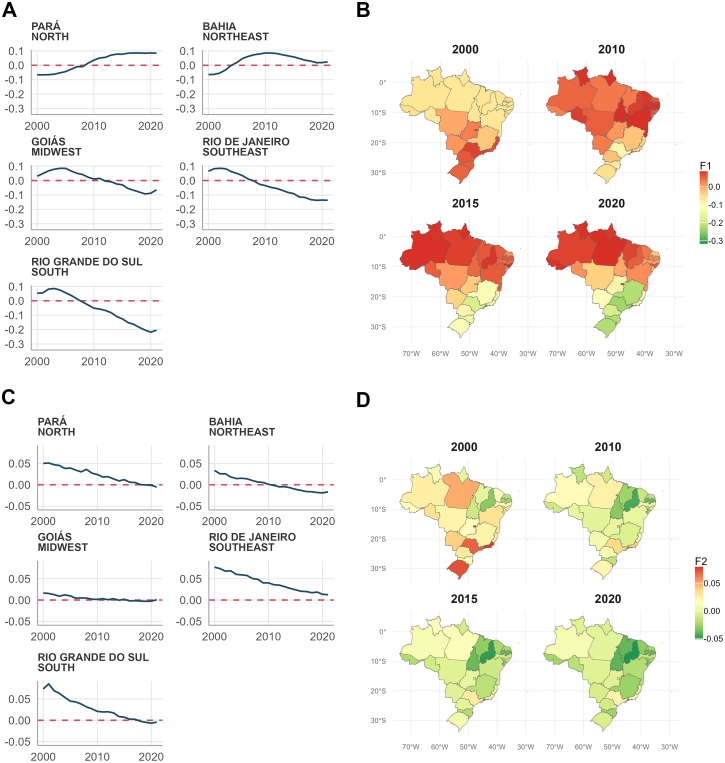
Contribution of the development index (factor one, or F1) and the Primary Health Care (PHC) index (factor two, or F2) to the predicted age-specific mortality rate (ASMR) for each Brazilian state from 2000 to 2021. **(A)** Contribution of the development index in a graphic representation for five Brazilian states, representing the country’s five macroregions, from 2000 to 2021. **(B)** Contribution of the development index for each state in a map representation for the years 2000, 2010, 2015 and 2020. **(C)** Contribution of the PHC index in a graphic representation for five Brazilian states, representing the country’s five macroregions, from 2000 to 2021. **(D)** Contribution of the PHC index for each state in a map representation for the years 2000, 2010, 2015 and 2020.

The *development index* (or factor one) increased over the study period across all states (Fig. 3A), showing negative values especially for Acre, Maranhão, Roraima and Alagoas in the years 2000–2010, and positive values for especially for Distrito Federal, São Paulo, Santa Catarina and Minas Gerais in the later years 2010–2020 (Supplementary Table S5). The association between the development index and mortality exhibited a non-linear pattern. In settings with lower socioeconomic conditions (development index < 0), increases in the index were associated with a progressively stronger positive effect on mortality, reaching a maximum at the index value of 0. Beyond this point, in more socioeconomically developed settings (development index > 0), further increases in the index were associated with a decreasing effect on mortality, eventually transitioning to a negative association. This non-linear effect translated across states such that LL mortality was rising over time in less developed states, when the development index was approaching zero, e.g., in the North and Northeast, while it is decreasing in the states of Midwest, Southeast and South, where the development index was positive and further increased (Fig. 4A and B).

The *Primary Health Care index* also increased over time across states in an almost opposite manner compared to the *development index*. States which had low values for the development index, had high values for the PHC index, e.g., Piauí, Tocantins, Maranhão and Paraíba. The increase of the PHC index was associated with an overall reduction in LL mortality (β = −0.03, 95% CI: −0.08 to 0.03, p = 0.40). While this decreasing effect was observed early within the study period in Northeastern states, only three of the South and Southeast states experienced it, namely, Minas Gerais, Santa Catarina, and Rio Grande do Sul (Fig. 4C and D). Although the *PHC index* term did not obtain statistical significance, it was kept in the final model considering the importance of its interpretation. It is important to note that the effect of factors on the predicted ASMR is understood and reported inside an additive model logic, and causality cannot be confirmed by this statistical approach.

## Discussion

Consistent with trends of many LMIC, our findings showed that Brazil did not follow the global decline in LL mortality, suggesting persistent healthcare inequities between our country and other HICs.^4^^,^^8^, ^9^, ^10^, ^11^, ^12^ While previous national studies reported declining or stable mortality trends for pediatric leukemias in earlier years in Brazil,^21^^,^^35^^,^^36^ our analysis of LL between 2000 and 2021 revealed increasing mortality rates. In the sense of regional disparities, our results showed higher mortality increases in less favourable socioeconomic contexts. These findings align with earlier reports, which also showed higher mortality increases in socioeconomically disadvantaged settings.^19^^,^^35^^,^^36^

In this study, we show that North and Northeast regions exhibited poorer outcomes for pediatric LL. This same regional pattern has been previously described by Ribeiro et al. (2007) for all leukemia subtypes in the Brazilian pediatric population from 1980 to 2002, reflecting long-standing socioeconomic inequalities across Brazil. The North and Northeast regions have historically exhibited poorer socioeconomic conditions, with lower Human Development Index (HDI) and Gross Domestic Product (GDP), compared to Brazil’s economically dominant South and Southeast regions.^37^^,^^38^ Although the country has undergone substantial socioeconomic improvements since 2000, reflected in the steady increase of the HDI across all 27 states, structural disparities remain evident, with the Southeast region maintaining economic dominance despite gradual decentralization.^17^^,^^37^^,^^38^

The development index (factor one) captured this dynamic, presenting a consistent increase over the study period, and showing a significant association with pediatric lymphoid leukemias mortality with regional disparities. We interpreted this index as an almost-spontaneous socioeconomic development gradient, analogous to established development indices such as the HDI, but additionally reflecting health system capacity at a more specialized and hospital-centred level. It captures multiple dimensions (e.g., the epidemiological transition, socioeconomic vulnerability, physician workforce distribution, and the quality of health data records in Brazil), and may serve as a health-adjusted development index suitable as a monitoring tool in future studies within the Brazilian context. In the North and Northeast regions, the increase of the development index was associated with a progressively stronger increase in predicted LL mortality, suggesting that poorer socioeconomic conditions may coincide with rising mortality. This suggests that these regions may be undergoing an earlier phase of the epidemiological transition, marked by a more recent shift from infectious to chronic-degenerative causes of death, coupled with still-developing registration systems, leading to an observable rise in diagnosed cases while structural limitations in the healthcare system constrain timely referral and access to effective treatment.^39^ In these contexts, improvements in diagnostic capacity and healthcare infrastructure coincide with higher mortality. In contrast, states in the South, Southeast, and Midwest displayed a declining association between the *development index* and LL mortality, and, in the South and Southeast regions, between 2008 and 2010, this index transitions its association with mortality from positive to negative. This indicates that once a certain structural threshold is achieved, improvements in socioeconomic conditions and healthcare capacity may begin to translate into better outcomes for pediatric LL.

In contrast, we did not find a significant association between the *Primary Health Care (PHC) index* (factor two) and the predicted pediatric LL mortality, despite the PHC’s expansion over the past two decades through the Family Health Strategy (FHS), the main federal policy guiding PHC development in Brazil.^40^ Although defined at the national level, the FHS is implemented and managed locally by municipalities, which results in heterogeneous expansion patterns across states. As a policy designed to prioritize vulnerable populations, its expansion occurred earlier and reached higher levels in regions with poorer socioeconomic indicators, resulting in a negative association with HDI.^40^ The lack of association between this covariate and the outcome in our analysis may be related to the relatively low number of deaths, given the rarity of these diseases. Previous studies have highlighted regional disparities in access to pediatric oncology care and their relationship with primary healthcare performance.^27^ Our findings suggest that, for pediatric LL, factors related to specialized care (e.g., treatment capacity, diagnostic precision) and socioeconomic development are more strongly associated with mortality than primary care coverage alone. It is also possible that PHC coverage does not necessarily reflect the level of professional training in oncologic recognition in primary care or population-level health literacy regarding leukemia symptoms, both of which may influence PHC effectiveness and contribute to the lack of a significant association observed. However, this hypothesis could not be directly evaluated within the scope of this study. Further research is needed to disentangle whether the absence of association is driven by PHC-related factors or by data limitations. Nevertheless, primary healthcare remains essential for early cancer detection and may be associated with improved outcomes when integrated with other health system components.^27^

The PHC assistance effectiveness and coverage in Brazil cannot be completely untied from the economic and health market development, but the logic behind these processes is different. While economic development follows almost-spontaneous trends, the Family Health Strategy was a deliberate governmental policy targeting vulnerable populations. The PCA analysis and the component structure support our interpretation. Physician and oncologist density were clustered within component one, alongside socioeconomic and demographic-related characteristics, while variables related to primary healthcare organization were grouped within component two. While physician and oncologist availability reflect specialized and tertiary care capacity, primary healthcare coverage represents community-based access and territorial reach. We see that some variables contributed to both components with similar weights. Rurality, for instance, contributed to both components in different directions. This reinforces the dual role of this variable, serving as a marker of both reduced access to specialized care (negative weight in the development index) and increased reliance on primary healthcare services (positive weight in the PHC index). This translates to rurality being smaller in contexts of higher values of development index, and being higher in contexts of higher PHC index. The contribution of HIV-related mortality in young children to both components should be interpreted as a contextual marker within the PCA structure rather than as evidence of a direct relationship with pediatric LL mortality.

Improvements in health data quality and diagnostic classification have also likely contributed to the observed increase in lymphoid leukemia mortality. Advances in healthcare infrastructure, including enhanced professional training and expanded availability of diagnostic services, can improve diagnostic precision, which is also reflected in the decreasing proportion of deaths by ill-defined causes. A previous Brazilian national scoping review reported a 68.5% reduction in “garbage codes” between 2000 and 2015, i.e., deaths that cannot be attributed to a valid underlying cause because they represent ill-defined conditions that don’t adequately explain the true cause of death, reflecting major improvements in death certification practices and cause-of-death coding.^41^ As a result, deaths that might previously have been recorded under unspecified cell-type leukemias or ill-defined categories may now be correctly classified as LL. However, these improvements have not occurred uniformly across the country and persistent regional and socioeconomic inequalities in mortality records remain evident, with ill-defined deaths occurring up to three times more frequently in municipalities with lower HDI scores.^42^^,^^43^ This is also reflected in the dataset of this present study. Greater variability in mortality estimates, including recurrent zero counts in some states (Fig. 2), likely reflects heterogeneity in data quality, diagnostic capacity, and small population sizes rather than true epidemiological instability.^37^^,^^42^ These year-to-year fluctuations are more evident in the North region, with five of the seven states facing this issue: Rondônia, Roraima, Acre, Amapá and Tocantins. These states also have smaller pediatric population sizes and lower numbers of pediatric lymphoid leukemia deaths compared to other Brazilian states (Supplementary Table S6), so that even minor variations can lead to exaggerated proportional fluctuations. As a consequence, in these settings with small populations and low annual case counts, any incomplete reporting may generate recurrent zero values, a well-recognized issue in analyses based on incomplete or sparse data.^31^ These limitations are inherent to ecological analyses using secondary data and should be interpreted within the broader context of structural inequalities in health information systems. Model parameters are reported for all states in Supplementary Table S2.

Although improvements in data quality and diagnostic capacity may partially explain the observed trends, they are unlikely to fully account for the higher mortality rates presented in our results.^26^ Mortality rates are shaped by both incidence and survival, and global LL mortality disparities are more strongly driven by survival rather than incidence differences.^39^^,^^44^ For instance, despite the higher leukemia incidence in North America compared to Latin America and the Caribbean (Age-specific incidence rate = 5.2 vs. 4.8/100,000), mortality remains substantially lower in North America (ASMR = 0.5 vs. 2.0/100,000), suggesting a strong influence of treatment access and effectiveness over the disease outcome.^45^ Additional factors, including genetic ancestry, may also play a role in these disparities. Higher mortality and relapse rates for LL were reported among Black and Hispanic children, raising the hypothesis that genomic ancestry-related factors may contribute to poorer prognosis in these populations. Nevertheless, Black and Hispanic people often live under worse socioeconomic conditions due to historical marginalization. Thus, these higher rates may relate to socioeconomic disadvantages, other than genomic factors.^46^^,^^47^ Brazil’s highly admixed population adds complexity to ancestry-based analyses. Admixture events reflect colonial and migration history, resulting in varying ancestry by region: predominantly European in South and Southeast, African in Northeast and Native-American in North.^48^ Comprehensive, state-level genomic data for the Brazilian population is only recently becoming available, and skin color has been shown to be an unreliable predictor of genetic ancestry.^46^^,^^49^ A recent work conducted by Nunes et al. (2025) identified 8.7 million previously unknown genetic variants in the Brazilian population and estimating the genetic composition of Brazilian states using genomic sequencing. Future investigations integrating genomic data could clarify genetic influences on pediatric LL outcomes, as it is not yet universally accepted that socioeconomic features can explain entirely the survival differences found in different ethnic groups regarding pediatric cancer.^50^

### Limitations

There is a lack of variables that directly assess treatment effectiveness, such as population- or state-level LL survival rates, which would provide a more comprehensive understanding of outcomes. Missing data, a common issue in epidemiological research, even when addressed, may introduce bias or reduce the robustness of certain analyses. Individual information on gender, race and ethnicity were not accessed in this study, and age groups were not stratified by sex due to the small number of cases. Therefore, we could not compare outcomes between sexes, genders, races or ethnicities. Finally, as an ecological study, it cannot infer causality or individual-level outcomes. Instead, the results should be interpreted as hypothesis-generating, aimed at identifying broader patterns and trends that warrant further investigation. To achieve more causal interpretations, study designs that better exploit temporal and spatial variation in exposure are needed (e.g., interrupted time series, regression discontinuity, or stepped-wedge designs). Additionally, observational studies specifically designed for causal inference using patient-level data would further strengthen the evaluation of these relationships.

### Conclusion

This study reveals an increasing mortality from lymphoid leukemias among Brazilian children and adolescents from 2000 to 2021, with heterogeneous regional patterns and sharper mortality rises in states from the North and Northeast regions. Regional disparities in mortality between Brazilian states were associated with structured socioeconomic, health-related and demographic characteristics. The growth of the economy and physician workforce capacity, the data quality improvement and the demographic structure of Brazilian states (represented by the *development index*) demonstrated a dynamic, non-linear association with model-estimated mortality rates. In less socioeconomically favoured settings (development index <0), the increase of this index was associated with an increase in pediatric lymphoid leukemias mortality. Conversely, in settings under better socioeconomic conditions (development index >0), the increase of this index shifted its association with mortality, eventually coinciding with reduced rates. The development index retained its modelled association with lymphoid leukemias mortality even in contexts of high primary health coverage. This observation, alongside the lack of a significant association between the Primary Health Care index and the outcome, suggests that expanding health access alone may be insufficient to overcome structural inequalities. The development index adds a new dimension to established socioeconomic measures, such as the Human Development Index (HDI). It may also serve as a health-adjusted development index for monitoring purposes, capturing aspects of the epidemiological transition, socioeconomic vulnerability, physician workforce, and health data quality in Brazil. Although this ecological analysis does not establish causality and does not account for treatment quality, individual-level characteristics, or genomic factors, it highlights persistent regional gradients in model-estimated mortality that warrant further investigation using patient-level and registry-based designs. Future studies should examine diagnostic timeliness, treatment access, and survival outcomes to better elucidate mechanisms underlying these disparities, thereby enabling stronger causal inference and informing targeted interventions.

## Contributors

BSS and MGPL conceived the study. BSS, UB, and MGPL developed the theoretical framework and contributed to the study design. BSS, CDEB, and ILO collected the data. BSS, UB, and MGPL analyzed the data and produced the visualizations. BSS, NLD, UB, and MGPL drafted the original manuscript. BSS, CDEB, ILO, NLD, UB, and MGPL reviewed and edited the manuscript. BSS and MGPL accessed and verified the underlying data. All authors had full access to the data reported in the study, provided critical input, approved the final version of the manuscript, and were responsible for the decision to submit the manuscript.

## Data sharing statement

All data used in this study are publicly available and were obtained from Brazilian governmental open-access databases. No individual participant data were collected or generated for this study. Aggregated state-level mortality data are available from the Department of Informatics of the Unified Health System (DATASUS; https://datasus.saude.gov.br/informacoes-de-saude-tabnet/, accessed Dec 22, 2025) and are sourced from the Mortality Information System (SIM). Population estimates are available from the Brazilian Institute of Geography and Statistics (IBGE; https://www.ibge.gov.br/estatisticas/downloads-estatisticas.html, accessed Dec 22, 2025). Socioeconomic and health system indicators are available from DATASUS, IBGE, and the Primary Health Care Secretariat (SAPS; https://relatorioaps.saude.gov.br/cobertura/aps, accessed Dec 22, 2025). A data dictionary is provided by each respective data source and variable in the supplementary material. No additional related documents (such as a study protocol, statistical analysis plan, or informed consent forms) are available, as this study exclusively used secondary, publicly available data. The data are available with publication and can be accessed without restriction through the official websites of the respective institutions. No approval process or data access agreement is required for data access.

## Editor note

The Lancet Group takes a neutral position with respect to territorial claims in published maps and institutional affiliations.

## Declaration of interests

BSS reports support from the Conselho Nacional de Desenvolvimento Científico e Tecnológico (CNPq, MCTI, Brazil; grant 406484/2022-8, INCT BioOncoPed), Coordenação de Aperfeiçoamento de Pessoal de Nível Superior (CAPES, Brazil), Fundação Carlos Chagas Filho de Amparo à Pesquisa do Estado do Rio de Janeiro (FAPERJ, Brazil), and the International Society of Paediatric Oncology (SIOP). CDEB and ILO report support from Fundação Carlos Chagas Filho de Amparo à Pesquisa do Estado do Rio de Janeiro (FAPERJ, Brazil). MGPL reports support from the Conselho Nacional de Desenvolvimento Científico e Tecnológico (CNPq, MCTI, Brazil; grant 406484/2022-8, INCT BioOncoPed) and Fundação Carlos Chagas Filho de Amparo à Pesquisa do Estado do Rio de Janeiro (FAPERJ, Brazil) through the Cientista do Nosso Estado programme. NLD and UB declare no competing interests. No financial interests or personal relationships influenced the work reported in this paper.
