# Supplementary material for: Spatiotemporal analysis and socioeconomic determinants of pediatric lymphoid leukemias mortality in Brazil from 2000 to 2021: an ecological study
Source: Lancet Reg Health Am. 2026 Jul 9;62:101557. doi: 10.1016/j.lana.2026.101557 (PMC13377150; doi:10.1016/j.lana.2026.101557)
Supplement: Supplementary Appendix 1 and 2 [file mmc1.pdf]

SUPPLEMENTARY MATERIAL

Spatiotemporal analysis and socioeconomic determinants of pediatric lymphoid leukemias mortality in Brazil from 2000 to 2021: An ecological study

TABLE OF CONTENTS

Supplementary Appendix 1

FIGURE S1 Distribution of ill-defined death proportions in the pediatric population in Brazil (2000–2021)

TABLE S1 Annual percentage change (APC) for the crude mortality rate from lymphoid leukemias (2000–2021)

FIGURE S2 Overall trend of the age-specific mortality rate (ASMR) from lymphoid leukemias in Brazil (2000–2021)

TABLE S2 Root Mean Squared Error (RMSE) of the model for each Brazilian state (age group 0–4 years)

FIGURE S3 Correlation matrix of the covariates included in the Principal Component Analysis (PCA)

TABLE S3 Tested generalized additive mixed models (GAMM), component terms, AIC and R^2

TABLE S4 Socioeconomic, demographic and health system variables, their sources, and their classification according to the Three-Delay Model

TABLE S5 Values of the development index (factor one) and primary health care index (factor two) per state and year

TABLE S6 Total reported deaths per state and age group (2000–2021)

Supplementary Appendix 2

How was the Health Adjusted Development Index (HADI) created?

TABLE S7 HDI and HADI values for Pernambuco and Rio Grande do Norte (2000, 2010, 2021)

TABLE S8 Principal components derived from PCA and variable loadings

TABLE S9 GAMM sensitivity analysis including HDI and HADI simultaneously

Supplementary material

FIGURE 1 Distribution of ill-defined death proportions in the pediatric population in Brazil over time (2000-2021) visualized by boxplots. Red dashed line represents 6% of ill-defined deaths.

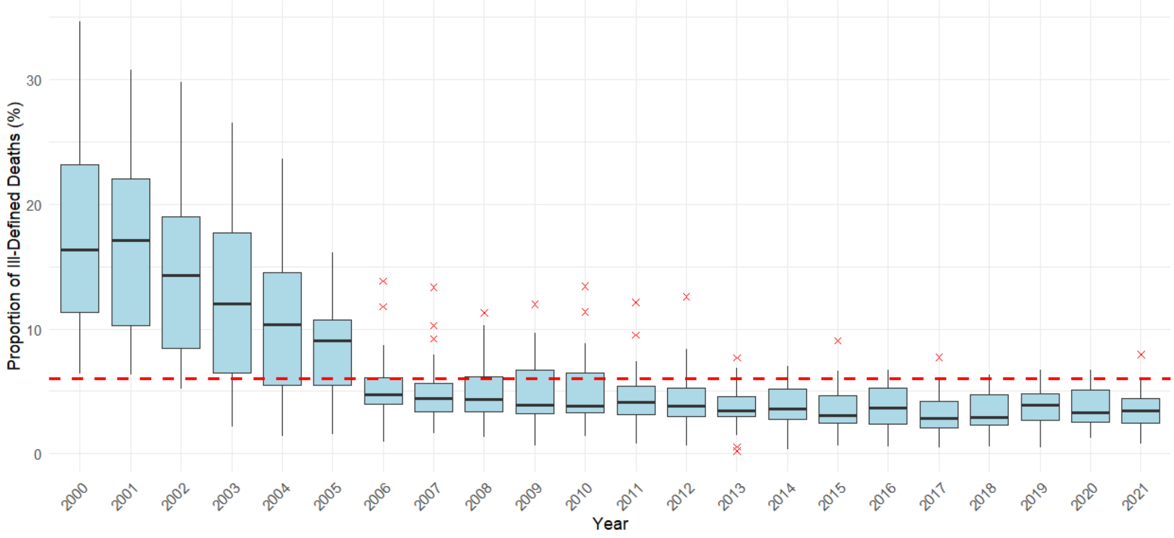

TABLE 1 Annual percentage change (APC) for the crude mortality rate (CMR) from lymphoid leukemias observed in the pediatric population (0–19 years) from 2000–2021 in Brazil and each of its five macroregions.

Region	APC	95%-CI[1]	p-value[2]	Trend
**North**	2·14	1·22, 3·22	<0·001*	Increase
**Northeast**	2·00	0·84, 3·28	0·002*	Increase
**South**	0·26	-0·67, 1·22	0·58	Stability
**Southeast**	-0·90	-1·55, -0·28	0·007*	Decrease
**Midwest**	0·36	-1·61, 2·47	0·67	Stability
**Total (Brazil)**	0·61	0·12, 1·12	0·014*	Increase

[1]CI = Confidence Interval; [2] = Wald test

FIGURE 2 Overall trend of the Age-specific mortality rate (ASMR) from lymphoid leukemias (LL) in Brazil (2000-2021) for age groups 0-4 years, 5-9 years, 10-14 years and 15-19 years.

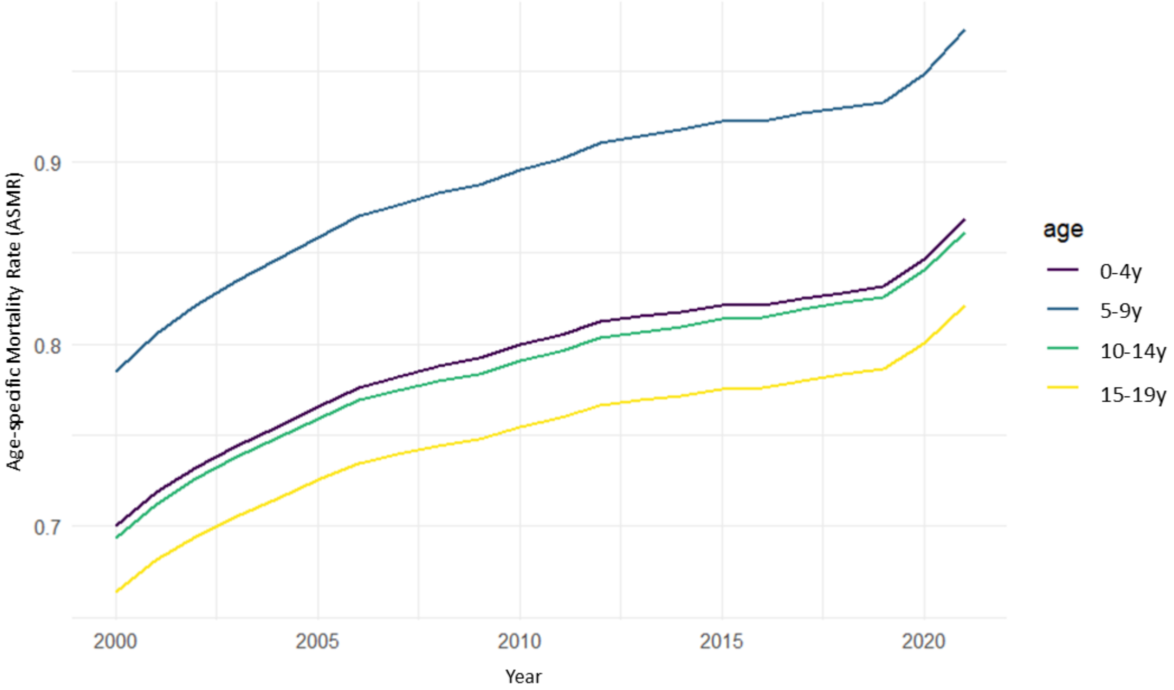

TABLE 2 Root Mean Squared Error (RMSE) of the model, mean crude mortality rate from lymphoid leukemias (mean CMR), and relative RMSE (RMSE divided by the mean CMR) for each Brazilian state in the age group of reference (0-4y). Pediatric population corresponds to the cumulative population aged 0-4y between 2000 and 2021 and total deaths, to the total number of deaths observed in this age group from 2000-2021.

State	RMSE	Mean CMR	Relative RMSE	Pediatric population (2000-2021)	Total deaths
**Roraima**	0·76	0·52	1·45	1,253,755	7
**Acre**	1·11	0·87	1·27	1,930,206	17
**Amapá**	0·89	0·86	1·03	1,753,407	15
**Distrito Federal**	0·54	0·54	1·00	4,893,468	26
**Mato Grosso do Sul**	0·69	0·72	0·96	4,631,836	33
**Tocantins**	0·81	0·96	0·85	2,902,986	28
**Rio Grande do Norte**	0·79	0·94	0·85	5,657,344	52
**Rondônia**	0·69	0·83	0·84	3,158,935	26
**Paraíba**	0·50	0·69	0·72	6,886,353	47

**Bahia**	0·37	0·51	0·72	25,214,867	128
**Espírito Santo**	0·60	0·84	0·72	6,103,204	51
**Sergipe**	0·60	0·84	0·71	3,963,042	33
**Pernambuco**	0·38	0·55	0·70	6,886,353	88
**Mato Grosso**	0·55	0·80	0·69	6,034,148	48
**Rio de Janeiro**	0·34	0·56	0·60	25,114,111	142
**Santa Catarina**	0·42	0·75	0·57	10,120,451	75
**Amazonas**	0·86	1·54	0·56	8,769,341	135
**Piauí**	0·69	1·30	0·54	5,937,255	75
**Goiás**	0·34	0·68	0·50	10,672,821	72
**Alagoas**	0·45	0·92	0·49	6,452,236	58
**Ceará**	0·45	0·92	0·48	15,460,574	141
**Maranhão**	0·47	1·03	0·46	14,429,837	148
**Pará**	0·43	1·01	0·43	16,982,158	173
**Rio Grande do Sul**	0·31	0·78	0·39	16,064,500	127
**Paraná**	0·30	0·80	0·38	17,455,399	139
**Minas Gerais**	0·25	0·66	0·37	30,943,446	208
**São Paulo**	0·15	0·69	0·22	68,116,798	469

FIGURE 3 Correlation matrix of the covariates included in the Principal Component Analysis (PCA).

Correlation Matrix[a]

		Life_expectancy	Fertility	Maternal_education	HIV_deaths_youger_five_years	Ill_defined_deaths_proportion	Rurality	Number_of_doctors	Number_of_oncologists	GDP	FHS_coverage	PHC_coverage
Correlation	Life_expectancy	1.000	-.591	.734	-.206	-.704	-.289	.559	.552	.597	.071	.169
	Fertility	-.591	1.000	-.648	.130	.661	.343	-.639	-.630	-.461	.029	.027
	Maternal_education	.734	-.648	1.000	-.223	-.777	-.562	.725	.704	.828	-.022	.068
	HIV_deaths_youger_five_years	-.206	.130	-.223	1.000	.100	.036	-.232	-.172	-.250	-.295	-.320
	Ill_defined_deaths_proportion	-.704	.661	-.777	.100	1.000	.373	-.499	-.464	-.521	-.030	-.093
	Rurality	-.289	.343	-.562	.036	.373	1.000	-.588	-.532	-.594	.474	.349
	Number_of_doctors	.559	-.639	.725	-.232	-.499	-.588	1.000	.932	.759	-.059	.030
	Number_of_oncologists	.552	-.630	.704	-.172	-.464	-.532	.932	1.000	.757	-.124	-.031
	GDP	.597	-.461	.828	-.250	-.521	-.594	.759	.757	1.000	-.192	-.091
	FHS_coverage	.071	.029	-.022	-.295	-.030	.474	-.059	-.124	-.192	1.000	.960
	PHC_coverage	.169	.027	.068	-.320	-.093	.349	.030	-.031	-.091	.960	1.000
Sig. (1-tailed)	Life_expectancy		.000	.000	.000	.000	.000	.000	.000	.000	.041	.000
	Fertility	.000		.000	.001	.000	.000	.000	.000	.000	.238	.254
	Maternal_education	.000	.000		.000	.000	.000	.000	.000	.000	.297	.048
	HIV_deaths_youger_five_years	.000	.001	.000		.007	.188	.000	.000	.000	.000	.000
	Ill_defined_deaths_proportion	.000	.000	.000	.007		.000	.000	.000	.000	.234	.011
	Rurality	.000	.000	.000	.188	.000		.000	.000	.000	.000	.000
	Number_of_doctors	.000	.000	.000	.000	.000	.000		.000	.000	.075	.232
	Number_of_oncologists	.000	.000	.000	.000	.000	.000	.000		.000	.001	.226
	GDP	.000	.000	.000	.000	.000	.000	.000	.000		.000	.013
	FHS_coverage	.041	.238	.297	.000	.234	.000	.075	.001	.000		.000
	PHC_coverage	.000	.254	.048	.000	.011	.000	.232	.226	.013	.000	

a. Determinant = 1.006E-005

Activate Windows

TABLE 3 Tested generalized additive mixed models (GAMM), the function of components one and two in the equation (absent – meaning, not included in the model –, linear or non-linear), the respective model's calculated AIC and R^2 and the statistical significance of component terms in the model. All models included, mandatorily, the offset of population, time (in years) and age groups (as factors) as additive and linear effects, and the spatial structure as a random effect.

GAM Model	Component one ("development index") term	Component two ("PHC index") term	Model AIC	Model R^2
**A**	Absent	Absent	4,739·8	0·751
**B**	Linear	Absent	4,732·5	0·758
**C**	Non-linear*	Absent	4,734·0	0·779
**D**	Absent	Linear	4,744·4	0·742
**E**	Absent	Non-linear	4,746·6	0·753
**F**	Linear	Linear	4,737·2	0·755
**G*** **	Non-linear*	Linear	4,738·6	0·779
**H**	Linear	Non-linear	4,740·9	0·762
**I**	Non-linear*	Non-linear	4,740·6	0·779

***Model of choice; *Statistical significance in the model ($p < 0·001$).

TABLE 4 Socioeconomic-related explanatory variables included in this study, their sources and their classification according to the different domains of the Three-Delay Model for Childhood Cancer Care.

Variable	Data Sources	Conceptual meaning	Stage – TDM	Stage – WHO GICC	Classification – SMH
**Life expectancy**	IBGE	Overall population health, sanitary improvement, demographic state	Reaching and receiving treatment	Onset of symptoms	Individual domain
**Crude Mortality Rate by HIV-related causes (0-5 years)**	DATASUS	Healthcare system performance, socioeconomic vulnerability	Reaching and receiving treatment	Onset of symptoms, diagnostic referral and treatment	Individual domain
**Crude mortality rate by ill-defined causes (0-19 years)**	DATASUS	Quality of health records, sanitary improvement	Reaching and receiving treatment	Onset of symptoms, referral and treatment	Individual domain
**Fertility rate**	IBGE	Demographic trends, urbanization, economic development	Seeking, reaching and receiving treatment	Onset of symptoms, diagnostic, referral and treatment	Interpersonal and family domain
**Maternal education**	IBGE	Socioeconomic vulnerability, health behaviours	Seeking and receiving treatment	Onset of symptoms, treatment and palliative care	Interpersonal and family domain
**Gross Domestic Product (GDP) per capita**	IBGE	Access to goods and services, economic development	Reaching and receiving treatment	Onset of symptoms, diagnostic, referral and treatment	Community and organization domain
**Number of doctors (per 1.000 habitants)**	CNES	Availability of medical care, health infrastructure and resources	Reaching and receiving treatment	Onset of symptoms, diagnostic and referral	Community and organization domain
**Number of oncologists (per 1.000 habitants)**	CNES	Capacity of diagnosis and specific treatment, infrastructure and resources	Receiving treatment	Diagnostic and treatment	Community and organization domain
**Primary health care assistance (PHC) coverage**	SAPS	Availability of basic healthcare services, healthcare access	Reaching treatment	Onset of symptoms, diagnostic and referral	Community and organization domain
**Family Health Strategy (FHS) coverage**	SAPS	Effective delivery of basic healthcare services	Reaching and receiving treatment	Onset of symptoms, diagnostic and referral	Community and organization domain
**Rurality rate**	IBGE	Healthcare access, socioeconomic vulnerability	Reaching and receiving treatment	Onset of symptoms, diagnostic referral and treatment	Community and organization domain

TDM – Three-Delay Model; WHO GICC – WHO Global Initiative for Childhood Cancer; SMH – Socioecological Model of Health. DATASUS – Information Technology Department of the Brazilian Unified Health System. CNES – Brazilian National Registry of Health Facilities. SAPS – Brazilian Primary Health Care Secretariat.

TABLE 5 Values of the development index (factor one) and primary health care (factor two) for each state and year.

State	Year	Factor one (development index)	Factor two (primary health care index)
**Rondônia**	2000	-1·94389	-1·3282
	2001	-1·94274	-1·30934
	2002	-1·5691	-0·94913
	2003	-1·55998	-1·25308
	2004	-1·23014	-0·722
	2005	-1·11121	-0·78768
	2006	-0·75261	-0·40438
	2007	-0·62206	-0·29638
	2008	-0·58914	-0·42961
	2009	-0·59015	-0·40233
	2010	-0·41095	-0·10197
	2011	-0·32946	-0·00833
	2012	-0·14122	0·107861
	2013	-0·03152	0·19663
	2014	-0·05993	-0·1231
	2015	0·206047	0·382919
	2016	0·320592	0·468073
	2017	0·314462	0·532215
	2018	0·421975	0·44136
	2019	0·558283	0·716447
	2020	0·46981	0·666088
	2021	0·244752	0·540536
**Acre**	2000	-2·54766	-0·53625
	2001	-2·47374	-1·15983
	2002	-2·1049	-0·61287
	2003	-1·8888	-0·19246
	2004	-1·85191	-0·16541
	2005	-1·45966	0·03243
	2006	-1·25587	0·145466

	2007	-1·13845	0·204697
	2008	-1·15025	-0·04214
	2009	-0·98048	0·02029
	2010	-0·82171	0·400219
	2011	-0·81807	0·457499
	2012	-0·7004	0·521851
	2013	-0·40689	0·666163
	2014	-0·35189	0·731798
	2015	-0·29631	0·788313
	2016	-0·1562	0·869122
	2017	-0·20797	0·568374
	2018	-0·12825	0·944001
	2019	-0·06213	1·009342
	2020	-0·12165	0·962372
	2021	-0·30151	0·899782
**Amazonas**	2000	-2·19026	-0·99031
	2001	-2·00877	-1·01849
	2002	-1·83622	-0·96429
	2003	-1·68699	-0·98767
	2004	-1·55417	-1·28229
	2005	-1·33691	-0·83592
	2006	-1·15748	-0·76251
	2007	-1·05459	-0·80411
	2008	-0·97704	-1·10977
	2009	-0·7791	-0·50912
	2010	-0·6607	-0·67569
	2011	-0·56995	-0·6415
	2012	-0·45135	-0·5347
	2013	-0·37312	-0·57093
	2014	-0·24299	-0·39164
	2015	-0·14835	-0·37261
	2016	-0·01149	-0·2734

	2017	0·032371	-0·26326
	2018	0·114826	-0·36995
	2019	0·24788	-0·1397
	2020	0·031638	-0·38188
	2021	-0·12321	-0·5554
**Roraima**	2000	-2·22436	-0·64142
	2001	-1·83172	-0·022
	2002	-1·19847	0·662004
	2003	-1·64138	-0·55061
	2004	-1·30179	-0·02903
	2005	-1·05404	0·558806
	2006	-0·93541	0·554222
	2007	-0·7423	0·592627
	2008	-0·80472	0·030872
	2009	-0·52989	0·571673
	2010	-0·2856	0·619554
	2011	-0·20545	0·603972
	2012	-0·30703	0·519485
	2013	-0·22644	0·498118
	2014	-0·1357	0·482578
	2015	-0·28321	-0·49087
	2016	-0·04622	0·411343
	2017	0·002784	0·378152
	2018	-0·15681	-0·09744
	2019	-0·07404	0·287277
	2020	-0·15861	0·141963
	2021	-0·30385	-0·00194
**Pará**	2000	-1·98388	-1·96904
	2001	-1·89354	-1·99966
	2002	-1·77297	-1·8484
	2003	-1·64722	-1·77199
	2004	-1·47696	-1·49964

	2005	-1·36856	-1·53889
	2006	-1·20965	-1·35313
	2007	-1·07292	-1·19027
	2008	-1·07315	-1·41665
	2009	-0·89608	-1·06672
	2010	-0·76258	-0·94464
	2011	-0·65702	-0·71456
	2012	-0·62212	-0·7427
	2013	-0·489	-0·5411
	2014	-0·41217	-0·35693
	2015	-0·4224	-0·46768
	2016	-0·29475	-0·22667
	2017	-0·24054	-0·20708
	2018	-0·21223	-0·0126
	2019	-0·11599	0·012905
	2020	-0·19181	0·049539
	2021	-0·12988	0·211017
**Amapá**	2000	-1·83946	-0·05123
	2001	-1·7161	-0·03761
	2002	-1·56028	0·002931
	2003	-1·27951	0·118445
	2004	-1·06284	0·188787
	2005	-0·8953	-0·11732
	2006	-0·44522	0·422839
	2007	-0·48527	0·027769
	2008	-0·47591	0·380053
	2009	-0·3078	0·420174
	2010	-0·28705	0·413866
	2011	-0·2442	0·414235
	2012	-0·24914	0·379433
	2013	-0·49234	-0·67819
	2014	-0·21252	0·374066

	2015	-0·17074	0·369246
	2016	-0·10614	0·055492
	2017	0·095384	0·445939
	2018	-0·02508	0·067148
	2019	-0·19853	-0·68378
	2020	-0·064	0·247775
	2021	-0·09174	0·215929
**Tocantins**	2000	-1·62764	0·446987
	2001	-1·50473	0·505143
	2002	-1·3004	0·607101
	2003	-1·10806	0·335906
	2004	-0·81705	0·858999
	2005	-0·76268	0·913983
	2006	-0·76014	0·363753
	2007	-0·53839	0·833091
	2008	-0·50247	0·871022
	2009	-0·41636	0·910569
	2010	-0·19265	1·216783
	2011	-0·30216	0·803368
	2012	-0·09463	1·08974
	2013	0·021136	1·146984
	2014	0·111186	1·404817
	2015	0·252566	1·472786
	2016	0·361749	1·51352
	2017	0·430066	1·551489
	2018	0·508451	1·58929
	2019	0·572428	1·613847
	2020	0·58189	1·580431
	2021	0·470099	1·469763
**Maranhão**	2000	-2·28662	-0·00341
	2001	-2·24603	-0·09176
	2002	-2·20239	0·105573

	2003	-2·08715	0·139658
	2004	-1·82782	0·286031
	2005	-1·53719	0·475554
	2006	-1·18717	0·716006
	2007	-1·06513	0·695138
	2008	-0·96598	0·775639
	2009	-0·86172	0·931675
	2010	-0·77926	0·997432
	2011	-0·78034	0·919912
	2012	-0·72529	1·008329
	2013	-0·65711	1·111546
	2014	-0·60796	1·174943
	2015	-0·57582	1·145984
	2016	-0·4518	1·348392
	2017	-0·44152	1·355418
	2018	-0·40372	1·470519
	2019	-0·3418	1·53649
	2020	-0·4523	1·522121
	2021	-0·46555	1·597121
**Piauí**	2000	-2·03883	1·211745
	2001	-1·89388	1·409949
	2002	-1·77431	1·354373
	2003	-1·50682	1·534919
	2004	-1·29777	1·531667
	2005	-1·19001	1·580406
	2006	-0·85235	1·718736
	2007	-0·70516	1·760077
	2008	-0·60331	1·705781
	2009	-0·49373	1·732335
	2010	-0·4171	1·734243
	2011	-0·34927	1·861145
	2012	-0·26876	1·871808

	2013	-0·18318	1·882315
	2014	-0·11664	2·003991
	2015	-0·07899	1·91037
	2016	-0·01223	1·806042
	2017	0·085962	2·047798
	2018	0·11946	2·047139
	2019	0·164381	1·947623
	2020	0·194111	2·00282
	2021	0·116083	1·929872
**Ceará**	2000	-1·61091	-0·50432
	2001	-1·40173	-0·41643
	2002	-1·23737	-0·21878
	2003	-1·12725	-0·2547
	2004	-0·93681	-0·16456
	2005	-0·70669	0·105693
	2006	-0·48397	0·065419
	2007	-0·30718	0·330133
	2008	-0·17669	0·498773
	2009	-0·10258	0·530759
	2010	-0·06742	0·473521
	2011	0·011494	0·670392
	2012	0·039486	0·68534
	2013	0·080227	0·782472
	2014	0·124964	0·768857
	2015	0·185689	0·892817
	2016	0·260501	0·997449
	2017	0·295542	1·063438
	2018	0·317043	1·091313
	2019	0·45077	1·198811
	2020	0·33466	1·167198
	2021	0·313777	1·107023
**Rio Grande do Norte**	2000	-1·38667	0·618826

	2001	-1·24291	0·355379
	2002	-1·01861	0·703437
	2003	-0·74615	0·69921
	2004	-0·46587	0·892568
	2005	-0·30794	0·947281
	2006	-0·19061	0·76638
	2007	-0·03845	0·896197
	2008	0·017499	0·891776
	2009	0·1408	0·915776
	2010	0·167574	0·678832
	2011	0·25222	1·00983
	2012	0·236808	0·644824
	2013	0·300807	0·642726
	2014	0·452004	0·997974
	2015	0·441367	0·864768
	2016	0·544551	0·85882
	2017	0·604311	0·8665
	2018	0·630303	0·756664
	2019	0·74327	0·873263
	2020	0·763755	0·93795
	2021	0·758387	0·887323
**Paraíba**	2000	-1·8378	0·906988
	2001	-1·66439	0·745204
	2002	-1·40738	0·904063
	2003	-1·2336	0·804547
	2004	-0·92224	1·23277
	2005	-0·7432	1·150399
	2006	-0·46627	1·324501
	2007	-0·37364	1·332223
	2008	-0·34781	1·245838
	2009	-0·24799	1·434367
	2010	-0·12771	1·45233

	2011	-0·10693	1·363232
	2012	0·01718	1·472029
	2013	0·124829	1·498712
	2014	0·121041	1·316089
	2015	0·163144	1·496335
	2016	0·284714	1·516941
	2017	0·345576	1·447287
	2018	0·437794	1·565366
	2019	0·458044	1·465015
	2020	0·448654	1·50137
	2021	0·329168	1·315004
**Pernambuco**	2000	-1·79947	-0·72203
	2001	-1·62144	-0·69378
	2002	-1·38176	-0·67996
	2003	-1·27284	-0·62732
	2004	-1·01731	-0·40664
	2005	-0·82889	-0·23791
	2006	-0·5375	-0·10508
	2007	-0·47182	-0·27799
	2008	-0·32559	0·009512
	2009	-0·19327	0·145175
	2010	-0·16634	-0·16464
	2011	-0·03406	0·247591
	2012	0·033425	0·218031
	2013	0·149824	0·278853
	2014	0·206062	0·315925
	2015	0·251126	0·384852
	2016	0·346633	0·372555
	2017	0·469209	0·561364
	2018	0·536751	0·578834
	2019	0·601906	0·546702
	2020	0·513852	0·548713

	2021	0·530543	0·630474
**Alagoas**	2000	-2·22551	-0·07148
	2001	-2·14638	-0·04277
	2002	-1·92788	-0·0292
	2003	-1·75525	-0·03528
	2004	-1·51104	0·22403
	2005	-1·27536	0·164287
	2006	-0·98339	0·357375
	2007	-0·82744	0·498527
	2008	-0·76492	0·436455
	2009	-0·62437	0·469042
	2010	-0·61526	0·274605
	2011	-0·48356	0·603643
	2012	-0·42358	0·428474
	2013	-0·32449	0·464624
	2014	-0·36642	-0·04712
	2015	-0·1475	0·515005
	2016	-0·08789	0·408667
	2017	0·060083	0·686889
	2018	0·114478	0·824274
	2019	0·246273	0·858989
	2020	0·180226	0·766634
	2021	0·159653	0·631501
**Sergipe**	2000	-1·4408	0·259768
	2001	-1·24374	0·56832
	2002	-1·06929	0·642097
	2003	-0·9587	0·557482
	2004	-0·63797	0·693601
	2005	-0·49104	0·767978
	2006	-0·26033	1·006014
	2007	-0·11428	1·060444
	2008	-0·03743	1·085925

	2009	0·088847	1·130814
	2010	0·176838	1·155109
	2011	0·071473	0·832896
	2012	0·266009	1·188132
	2013	0·297653	1·059077
	2014	0·365456	1·224401
	2015	0·426977	1·252191
	2016	0·527961	1·282874
	2017	0·586688	1·319402
	2018	0·641765	1·348601
	2019	0·744671	1·393755
	2020	0·649318	1·31444
	2021	0·616351	1·131092
**Bahia**	2000	-2·07427	-1·31856
	2001	-1·6805	-1·0103
	2002	-1·49876	-1·01058
	2003	-1·25551	-0·71685
	2004	-1·01283	-0·57124
	2005	-0·87293	-0·59737
	2006	-0·68216	-0·55145
	2007	-0·56886	-0·38864
	2008	-0·46657	-0·25596
	2009	-0·39359	-0·21218
	2010	-0·1947	-0·03882
	2011	-0·15486	0·061767
	2012	-0·07011	0·192094
	2013	0·042233	0·175771
	2014	0·117374	0·276428
	2015	0·179491	0·421015
	2016	0·260812	0·516539
	2017	0·341706	0·625608
	2018	0·38576	0·644515

	2019	0·45595	0·71383
	2020	0·452618	0·749115
	2021	0·42009	0·649838
**Minas Gerais**	2000	-1·21696	-0·92902
	2001	-0·94583	-0·75031
	2002	-0·73524	-0·62783
	2003	-0·56977	-0·50976
	2004	-0·36334	-0·41763
	2005	-0·15859	-0·19101
	2006	0·079059	-0·17183
	2007	0·2538	-0·02961
	2008	0·436941	0·145202
	2009	0·591035	0·144151
	2010	0·731517	0·20458
	2011	0·856524	0·342127
	2012	0·956838	0·453772
	2013	1·112278	0·497276
	2014	1·24765	0·578863
	2015	1·388909	0·658259
	2016	1·560712	0·76068
	2017	1·663016	0·804321
	2018	1·815827	0·871553
	2019	1·953225	1·000841
	2020	2·032081	1·036956
	2021	1·957942	0·967187
**Espírito Santo**	2000	-0·47019	-1·2268
	2001	-0·27509	-1·04043
	2002	-0·08274	-0·94692
	2003	0·018978	-1·26331
	2004	0·147683	-1·10081
	2005	0·372334	-0·52833
	2006	0·412236	-0·69072

	2007	0·525876	-0·63108
	2008	0·579591	-0·58039
	2009	0·664962	-0·61926
	2010	0·839527	-0·23898
	2011	0·767287	-0·41498
	2012	0·791151	-0·56316
	2013	0·977973	-0·18231
	2014	1·061627	-0·12011
	2015	1·124817	0·019488
	2016	1·237356	-0·0199
	2017	1·26084	-0·06393
	2018	1·368431	0·179585
	2019	1·449911	0·234414
	2020	1·336298	0·162724
	2021	1·304145	0·138645
**Rio de Janeiro**	2000	-0·54323	-3·0231
	2001	-0·33392	-2·89249
	2002	-0·16156	-2·65854
	2003	-0·08019	-2·69172
	2004	0·146762	-2·36347
	2005	0·242041	-2·31523
	2006	0·353859	-2·26018
	2007	0·504812	-1·98192
	2008	0·59182	-1·90923
	2009	0·739887	-1·57641
	2010	0·799789	-1·58093
	2011	0·878577	-1·40134
	2012	0·927112	-1·37216
	2013	1·011351	-1·22454
	2014	1·10603	-1·07016
	2015	1·15982	-0·94117
	2016	1·307632	-0·80853

	2017	1·329735	-0·77813
	2018	1·424983	-0·6757
	2019	1·458298	-0·72823
	2020	1·447749	-0·52586
	2021	1·455652	-0·48909
**São Paulo**	2000	-0·43871	-2·71393
	2001	-0·17255	-2·47632
	2002	0·045021	-2·25402
	2003	0·223439	-2·03858
	2004	0·395089	-1·91559
	2005	0·544855	-1·82216
	2006	0·699408	-1·74207
	2007	0·824788	-1·64364
	2008	0·955904	-1·63142
	2009	1·092781	-1·52394
	2010	1·20534	-1·45495
	2011	1·315468	-1·34243
	2012	1·403854	-1·31672
	2013	1·539919	-1·20646
	2014	1·649262	-1·11194
	2015	1·759312	-1·06461
	2016	1·857954	-1·01543
	2017	1·970316	-0·93621
	2018	2·075542	-0·8763
	2019	2·172324	-0·84248
	2020	2·176941	-0·86072
	2021	2·109381	-0·91752
**Paraná**	2000	-0·58274	-1·15582
	2001	-0·37888	-1·11312
	2002	-0·21528	-0·97124
	2003	-0·07252	-0·79374
	2004	0·064808	-0·6858

	2005	0·121419	-0·7627
	2006	0·323216	-0·51412
	2007	0·461661	-0·41702
	2008	0·52669	-0·37309
	2009	0·638154	-0·31174
	2010	0·71299	-0·32722
	2011	0·835572	-0·13021
	2012	0·905662	-0·17754
	2013	0·997687	-0·11362
	2014	1·112295	0·050537
	2015	1·204846	0·042534
	2016	1·356341	0·113851
	2017	1·460162	0·203802
	2018	1·557646	0·228372
	2019	1·69731	0·357962
	2020	1·75772	0·367706
	2021	1·56536	0·207669
**Santa Catarina**	2000	-0·61184	-0·45107
	2001	-0·45395	-0·42975
	2002	-0·28931	-0·31979
	2003	-0·10799	-0·44311
	2004	0·122696	0·130952
	2005	0·154719	-0·08976
	2006	0·466432	0·270759
	2007	0·588684	0·309392
	2008	0·753033	0·436564
	2009	0·911705	0·500423
	2010	1·044016	0·611318
	2011	1·091073	0·582403
	2012	1·170407	0·569904
	2013	1·307521	0·631302
	2014	1·443869	0·74735

	2015	1·530278	0·739109
	2016	1·697244	0·907083
	2017	1·800088	0·952047
	2018	1·877737	0·94166
	2019	1·998454	1·040902
	2020	2·041681	0·995139
	2021	1·933606	0·944931
**Rio Grande do Sul**	2000	-0·64641	-2·88043
	2001	-0·61969	-3·34583
	2002	-0·35757	-2·72189
	2003	-0·1865	-2·46912
	2004	0·026011	-2·0962
	2005	0·199331	-1·75351
	2006	0·333206	-1·69551
	2007	0·487655	-1·49898
	2008	0·629	-1·22946
	2009	0·766835	-1·04393
	2010	0·905766	-0·81837
	2011	0·942528	-0·76331
	2012	1·007829	-0·78222
	2013	1·103282	-0·67975
	2014	1·277596	-0·34312
	2015	1·383945	-0·27708
	2016	1·549941	-0·09846
	2017	1·629616	-0·08804
	2018	1·781854	0·093735
	2019	1·916567	0·191979
	2020	2·031379	0·273814
	2021	1·923339	0·15961
**Mato Grosso do Sul**	2000	-0·9308	-1·56559
	2001	-0·545	-0·76915
	2002	-0·50049	-1·21331

	2003	-0·33301	-0·99702
	2004	-0·189	-0·65321
	2005	-0·12078	-0·59394
	2006	0·034107	-0·51915
	2007	0·113301	-0·60097
	2008	0·209154	-0·2657
	2009	0·321702	-0·20471
	2010	0·357402	-0·28445
	2011	0·473298	-0·08913
	2012	0·55557	-0·15161
	2013	0·669311	-0·08549
	2014	0·725332	-0·03601
	2015	0·83059	0·132739
	2016	0·91607	0·174009
	2017	1·027195	0·241307
	2018	1·140179	0·300824
	2019	1·204743	0·215634
	2020	1·253885	0·32002
	2021	1·1209	0·190937
**Mato Grosso**	2000	-1·02963	-0·87149
	2001	-0·93511	-0·84557
	2002	-0·73699	-0·43554
	2003	-0·66763	-0·70021
	2004	-0·63643	-0·74792
	2005	-0·48771	-0·37196
	2006	-0·25268	-0·35935
	2007	-0·05532	-0·17742
	2008	-0·0229	-0·25021
	2009	0·058355	-0·118
	2010	0·20407	0·047664
	2011	0·211798	0·061597
	2012	0·255084	-0·10972

	2013	0·40462	0·148105
	2014	0·420512	0·165338
	2015	0·523983	0·121328
	2016	0·661198	0·165453
	2017	0·713531	0·112494
	2018	0·85279	0·339213
	2019	0·989424	0·387728
	2020	0·805746	0·148062
	2021	0·762829	0·063088
**Goiás**	2000	-0·79816	-0·64293
	2001	-0·67275	-0·60633
	2002	-0·51914	-0·49637
	2003	-0·39252	-0·35508
	2004	-0·26683	-0·4769
	2005	-0·14789	-0·37894
	2006	0·114993	-0·16931
	2007	0·241234	-0·19277
	2008	0·299568	-0·17478
	2009	0·429139	-0·07914
	2010	0·503127	-0·05727
	2011	0·475466	-0·12892
	2012	0·599543	-0·02627
	2013	0·625763	-0·12561
	2014	0·725669	0·017154
	2015	0·766373	-0·06681
	2016	0·930881	0·084237
	2017	1·011538	0·064123
	2018	1·092336	0·093002
	2019	1·172744	0·116977
	2020	1·142431	0·107934
	2021	0·986814	-0·02831
**Distrito Federal**	2000	-0·10445	-3·13066

	2001	0·037478	-2·80502
	2002	0·179588	-3·00251
	2003	0·470572	-2·49625
	2004	0·591119	-2·60409
	2005	0·776893	-2·24245
	2006	0·870201	-2·35674
	2007	1·057739	-2·12852
	2008	1·202668	-1·89527
	2009	1·299876	-1·79212
	2010	1·41363	-1·80883
	2011	1·65037	-1·55423
	2012	1·749047	-1·5699
	2013	1·918428	-1·33502
	2014	2·103212	-1·21694
	2015	2·213546	-1·23647
	2016	2·442001	-1·11267
	2017	2·621232	-0·88394
	2018	2·823356	-0·77329
	2019	3·039084	-0·66209
	2020	3·084665	-0·68705
	2021	3·149753	-0·69057

TABLE 6 Total reported deaths per state and age group during the entire study period (2000-2021).

State	Age group	Number of deaths
**North**		
**Rondônia**	0-4y	26
	5-9y	32
	10-14y	22
	15-19y	17
**Acre**	0-4y	17
	5-9y	27
	10-14y	15

	15-19y	21
**Amazonas**	0-4y	135
	5-9y	107
	10-14y	86
	15-19y	78
**Roraima**	0-4y	7
	5-9y	11
	10-14y	9
	15-19y	8
**Pará**	0-4y	173
	5-9y	217
	10-14y	164
	15-19y	133
**Amapá**	0-4y	15
	5-9y	16
	10-14y	23
	15-19y	9
**Tocantins**	0-4y	28
	5-9y	21
	10-14y	20
	15-19y	27
**Northeast**		
**Maranhão**	0-4y	148
	5-9y	131
	10-14y	121
	15-19y	98
**Piauí**	0-4y	75
	5-9y	88
	10-14y	59
	15-19y	50
**Ceará**	0-4y	141
	5-9y	187

	10-14y	148
	15-19y	131
**Rio Grande do Norte**	0-4y	52
	5-9y	63
	10-14y	48
	15-19y	66
**Paraíba**	0-4y	47
	5-9y	48
	10-14y	52
	15-19y	47
**Pernambuco**	0-4y	88
	5-9y	102
	10-14y	100
	15-19y	116
**Alagoas**	0-4y	58
	5-9y	49
	10-14y	53
	15-19y	47
**Sergipe**	0-4y	33
	5-9y	30
	10-14y	37
	15-19y	38
**Bahia**	0-4y	128
	5-9y	159
	10-14y	160
	15-19y	165
**Southeast**		
**Minas Gerais**	0-4y	208
	5-9y	234
	10-14y	239
	15-19y	210
**Espírito Santo**	0-4y	51

	5-9y	58
	10-14y	66
	15-19y	47
**Rio de Janeiro**	0-4y	142
	5-9y	231
	10-14y	205
	15-19y	223
**São Paulo**	0-4y	469
	5-9y	571
	10-14y	534
	15-19y	545
**South**		
**Paraná**	0-4y	139
	5-9y	169
	10-14y	176
	15-19y	154
**Santa Catarina**	0-4y	75
	5-9y	94
	10-14y	80
	15-19y	103
**Rio Grande do Sul**	0-4y	127
	5-9y	154
	10-14y	139
	15-19y	193
**Midwest**		
**Mato Grosso do Sul**	0-4y	33
	5-9y	51
	10-14y	28
	15-19y	37
**Mato Grosso**	0-4y	48
	5-9y	45
	10-14y	53

	15-19y	46
**Goiás**	0-4y	72
	5-9y	107
	10-14y	101
	15-19y	110
**Distrito Federal**	0-4y	26
	5-9y	34
	10-14y	41
	15-19y	35

SUPPLEMENTARY APPENDIX 2

How was the Health Adjusted Development Index (HADI) created?

The Human Development Index (HDI) (1) captures important dimensions of socioeconomic development and is a key indicator both in health economics and epidemiological analysis. However, it may not fully explain the inequalities observed in pediatric lymphoid leukemia outcomes in Brazil, since these inequalities are unlikely driven by socioeconomic differences alone.

As an illustration, states with similar HDI values, such as Pernambuco and Rio Grande do Norte, present very different patterns in the variance between observed mortality rates in each year (Figure 2 in the main text), highlighting underlying factors involved in this distribution that cannot be explained solely by the HDI.

TABLE 7 Values of the Human Development Index (HDI) and the Health Adjusted Development Index (HADI) for the states Pernambuco and Rio Grande do Norte (northeastern states) in the years 2000, 2010 and 2021.

**State**	**Year 2000**		**Year 2010**		**Year 2021**	
	**HDI**	**HADI**	**HDI**	**HADI**	**HDI**	**HADI**
**Pernambuco**	0·544	-1·799	0·673	-0·166	0·719	0·531
**Rio Grande do Norte**	0·552	-1·387	0·684	0·168	0·728	0·758

In this context, the Health Adjusted Development Index (HADI), in the main article also referred to as the "development index", emerged in this study as a consequence of our analytical process rather than a predefined construct.

As we sought to understand whether socioeconomic conditions alone could explain pediatric lymphoid leukemia mortality disparities across Brazilian states, we aimed to construct a more accurate and contextually richer index, one more suitable to describe the specific questions we were asking. While the HDI has been associated with pediatric leukemia mortality in previous Brazilian literature, several variables correlated with pediatric cancer outcomes in international literature are not captured by the HDI alone.

Construction of HADI

We combined socioeconomic state-level characteristics with variables reflecting health system organization, physician workforce, and health data quality, aiming to capture not only socioeconomic inequalities, but also structural disparities within the Brazilian health system. To avoid multicollinearity and to capture the conceptual interplay among these variables rather than their isolated effects, we applied dimensionality reduction through principal component analysis (PCA).

TABLE 8 Principal components derived from principal component analysis (PCA) and the corresponding loadings (weights) for each variable inside the components. Loadings represent the correlation between the original variables and each component, while squared loadings indicate the relative contribution of each variable to the component structure. Variables are grouped according to their highest loading to facilitate interpretation. For clarity, loadings with absolute values <|0.200| are not displayed.

	**Variables` loadings**	
	**Component one**	**Component two**
**Number of doctors**	0·899	
**Number of oncologists**	0·885	
**HDI**	0·878	
**Fertility rate**	-0·819	
**Proportion of ill-defined deaths**	-0·764	
**PHC Coverage**		0·968
**FHS Coverage**		0·960

HIV mortality (< 5y)	-0·234	-0·515

Validation of the HADI

In sensitivity analyses, HADI was evaluated against HDI by jointly including both variables in a Generalized Additive Mixed Model (GAMM). Despite their redundancy, both terms retained statistically significant associations with the outcome ($p < 0.05$). This finding revealed that HADI contributes additional, independent information, capturing a variance not fully explained by HDI alone.

TABLE 9 Association between model covariates and pediatric lymphoid leukemia mortality (GAMM, sensitivity analysis including HDI and HADI simultaneously).

Characteristic	Beta	95% CI[1]	p-value
Age groups			
0–4 years (reference)	—	—	
5–9 years	0·11	0·04, 0·19	0·002
10–14 years	-0·01	-0·08, 0·06	0·80
15–19 years	-0·06	-0·13, 0·02	0·12
Year	0·02	0·00, 0·03	0·031
Component one (HADI, smooth term)			0·049
HDI (smooth term)			<0·001
[1] CI = Confidence Interval			

We included the HDI dimensions: life expectancy, education (included as maternal education due to the pediatric cancer scope of this analysis), and gross domestic product (GDP) per capita. The resulting components of the PCA using these variables preserved the same aggregation structure as the analysis conducted with the HDI, suggesting a conceptual equivalence. This finding guided our final analytical choice: a structure that better captures Brazilian health dynamics, improves interpretability, and, most importantly, adds meaningful information to what was previously known about socioeconomic determinants of pediatric cancer mortality in Brazil.

The composition of HADI can be found in our main manuscript, Table 1, where it is called "development index". The conceptual meaning of each included variable can be also found in

the main manuscript, Figure 1. Values of HADI for each state and year can be found in the supplementary material, Table 5, where it is also called “development index”.

References

1. United Nations Development Programme. Human Development Index (HDI) [Internet]. 2026 [cited 2026 May 12]. Available from: https://hdr.undp.org/data-center/human-development-index#/indicies/HDI
